# Investigation of fitness cost, copy number variation and excision rate of Tn*916*/Tn*916*-like elements in oral streptococci and enterococci

**DOI:** 10.1080/20002297.2017.1325191

**Published:** 2017-06-09

**Authors:** Mohammed Al-Haroni, Tracy Lunde, Adam Roberts

**Affiliations:** ^a^ Department of Clinical Dentistry, Faculty of Health Sciences, UiT The Arctic University of Norway, Tromsø, Norway; ^b^ UCL Eastman Dental Institute, UCL, London, UK

## Abstract

The Tn916 and other related elements are broad host range conjugative transposons that are widely spread in bacteria. These elements contribute to the spread of antibiotic resistance genes (ARG) between bacterial species. Variation in the Tn*916*/Tn*916*-like elements copy number (Tn*916*CN) and their excision frequency (Tn*916*ExFr) from their host genome could play a role in propagation of ARG. In this study, the Tn*916*CN and the Tn*916*ExFr in oral streptococci and enterococci were determined using droplet digital PCR (ddPCR). In addition, we investigated the fitness cost associated with new acquisition of Tn*916* in a selected oral streptococci and enterococci strains. Amelioration of fitness cost and mechanism(s) to mitigate fitness loss were evaluated by ddPCR and next generation sequencing (NGS). We found the ddPCR is superior to southern blot to determine Tn*916*CN. It was also found that both the excision rate of Tn*916* and its autonomous replication increase because of antimicrobial challenge. Loss of bacterial fitness was usually associated with multiple acquisitions of Tn*916*. It is concluded that amelioration of fitness cost is achieved after a few hundred generations and by maintaining one copy of Tn*916* in bacterial genome under no selection pressure.

